# Comparison of seven CD19 CAR designs in engineering NK cells for enhancing anti‐tumour activity

**DOI:** 10.1111/cpr.13683

**Published:** 2024-06-03

**Authors:** Yao Wang, Jianhuan Li, Zhiqian Wang, Yanhong Liu, Tongjie Wang, Mengyun Zhang, Chengxiang Xia, Fan Zhang, Dehao Huang, Leqiang Zhang, Yaoqin Zhao, Lijuan Liu, Yanping Zhu, Hanmeng Qi, Xiaofan Zhu, Wenbin Qian, Fangxiao Hu, Jinyong Wang

**Affiliations:** ^1^ Guangzhou Institutes of Biomedicine and Health, Chinese Academy of Sciences Guangzhou China; ^2^ State Key Laboratory of Stem Cell and Reproductive Biology Institute for Stem Cell and Regeneration, Institute of Zoology, Chinese Academy of Sciences Beijing China; ^3^ Beijing Institute for Stem Cell and Regenerative Medicine Beijing China; ^4^ University of Chinese Academy of Sciences Beijing China; ^5^ GMU‐GIBH Joint School of Life Sciences Guangzhou Medical University Guangzhou China; ^6^ State Key Laboratory of Experimental Hematology & National Clinical Research Center for Blood Diseases Institute of Hematology & Blood Diseases Hospital, Chinese Academy of Medical Sciences & Peking Union Medical College Tianjin China; ^7^ Center for Stem Cell Medicine & Department of Stem Cell and Regenerative Medicine Chinese Academy of Medical Sciences & Peking Union Medical College Tianjin China; ^8^ Department of Hematology, the Second Affiliated Hospital, College of Medicine Zhejiang University Zhejiang Hangzhou China

## Abstract

Chimeric antigen receptor‐natural killer (CAR‐NK) cell therapy is emerging as a promising cancer treatment, with notable safety and source diversity benefits over CAR‐T cells. This study focused on optimizing CAR constructs for NK cells to maximize their therapeutic potential. We designed seven CD19 CAR constructs and expressed them in NK cells using a retroviral system, assessing their tumour‐killing efficacy and persistence. Results showed all constructs enhanced tumour‐killing and prolonged survival in tumour‐bearing mice. In particular, CAR1 (CD8 TMD‐CD3ζ SD)‐NK cells showed superior efficacy in treating tumour‐bearing animals and exhibited enhanced persistence when combined with OX40 co‐stimulatory domain. Of note, CAR1‐NK cells were most effective at lower effector‐to‐target ratios, while CAR4 (CD8 TMD‐OX40 CD‐ FcεRIγ SD) compromised NK cell expansion ability. Superior survival rates were noted in mice treated with CAR1‐, CAR2 (CD8 TMD‐ FcεRIγ SD)‐, CAR3 (CD8 TMD‐OX40 CD‐ CD3ζ SD)‐ and CAR4‐NK cells over those treated with CAR5 (CD28 TMD‐ FcεRIγ SD)‐, CAR6 (CD8 TMD‐4‐1BB CD‐CD3ζ 1‐ITAM SD)‐ and CAR7 (CD8 TMD‐OX40 CD‐CD3ζ 1‐ITAM SD)‐NK cells, with CAR5‐NK cells showing the weakest anti‐tumour activity. Increased expression of exhaustion markers, especially in CAR7‐NK cells, suggests that combining CAR‐NK cells with immune checkpoint inhibitors might improve anti‐tumour outcomes. These findings provide crucial insights for developing CAR‐NK cell products for clinical applications.

## Graphical abstract

The best chimeric antigen receptor (CAR) design in natural killer (NK) cell context remains elusive. Wang et al. reveal that a CD19 CAR design with CD8 TMD‐CD3ζ SD exhibits strong anti‐tumour activity. Further, combination with the OX40 CD into the CD19 CAR design enhances the persistence of CAR‐NK cells in vivo.

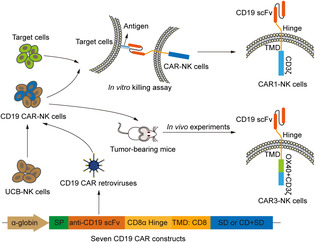

## 1 INTRODUCTION

Chimeric antigen receptors (CARs) are engineered to redirect immune cells, such as T cells,^1^ NK cells^2^ and macrophages,^3^ to target and eliminate cells expressing specific ligands. In particular, CAR‐T cells have shown remarkable success in cancer immunotherapy.^4^ A typical CAR consists of four modular components: an antigen‐binding domain, a hinge, a transmembrane domain (TMD) and an intracellular domain. The latter usually comprises a signalling domain (SD) and at least one co‐stimulatory domain (CD), both crucial for immune cell activation, proliferation and persistence.^5^

Currently, optimization efforts in CAR design are predominantly focused on CAR‐T cells. To boost their anti‐tumour efficacy, various intracellular domains, including different CDs (CD28, 4‐1BB, OX40, TLR2, etc.) and SDs (CD3ζ, CD3ζ variants, FcεRIγ, etc.), have been developed.^5^, ^6^, ^7^, ^8^, ^9^ Comparative data of different CDs in CAR‐T cells reveal their unique contributions to cell activation, proliferation and persistence.^10^, ^11^ One emphasis of improving the long‐term therapeutic efficacy of CAR‐T cells is to seek approaches to modify these cells to prolong their in vivo persistence.^12^ For example, OX40 has outperformed CD28 in proliferation and cytotoxicity, and 4‐1BB in persistence and proliferation of CAR‐T cells.^11^ The number of immune‐receptor‐tyrosine‐based activation motifs (ITAMs) in the intracellular domain determines the activation magnitude of CAR‐T cells.^13^ Conversely, reducing ITAMs, including omitting ITAM‐containing CDs or silencing ITAMs from CD3ζ, improves the survival and memory status of CAR‐T cells.^14^, ^15^ These findings underscore that subtle variations in CAR designs, especially in the intracellular domains, can significantly impact CAR‐T cell functionality.^16^, ^17^ However, excessive signalling from CARs can lead to adverse effects such as reduced proliferation, impaired survival, rapid exhaustion and high‐level tonic signalling.^18^, ^19^, ^20^

NK cells can directly destroy multiple tumour cells, eliminating the need for MHC‐neoantigen presentation, a mechanism utilized by T cells.^21^ Engineered CAR‐NK cells show enhanced anti‐tumour abilities without compromising their safety.^22^ Most CAR structures currently used in engineered CAR‐NK cells refer to those used in CAR‐T cells.^23^ 4‐1BB co‐stimulatory signalling has been found to enhance the long‐term anti‐tumour activity and persistence in NK cells.^24^ OX40 has also emerged as a suitable CD for CAR‐NK cells.^25^ However, the impact of ITAM numbers in the intracellular domain of CAR‐NK cells is still unclear. Due to the different features between NK cells and T cells, there are unique challenges in maintaining the proliferation, persistence and longevity of NK cells when equipped with CARs.^26^

In our study, we compared seven CAR designs in CD19 CAR‐NK cells by assembling TMDs of CD8 or CD28 molecules, CDs of 4‐1BB or OX40 molecules, and SDs with various numbers of ITAMs. We further modified the ITAMs by editing CD3ζ or replacing it with FcεRIγ.^14^, ^27^, ^28^, ^29^ Our data show that the CD8 TMD‐CD3ζ SD configuration exhibited the strongest anti‐tumour activity. Reducing ITAMs in CAR designs barely improves the therapeutic efficacy of CAR‐NK cells, contradicting the findings in CAR‐T cells.^14^ We observed an increase in the expression of exhaustion markers in all CAR‐NK cells, especially in CAR7 (CD8 TMD‐OX40 CD‐CD3ζ 1‐ITAM SD)‐NK cells, after extended engagement with targets. This observation suggests that combining CAR‐NK cells with immune checkpoint inhibitors could potentially enhance therapeutic outcomes. Our study provides crucial insights for the design of CAR‐NK cell products for clinical applications.

## 2 MATERIALS AND METHODS

### 2.1 Cell lines

Human embryonic kidney (HEK) 293T cells (ATCC) were cultured in Dulbecco's modified Eagle medium (Gibco) supplemented with 10% fetal bovine serum (FBS, HUANGKE). Human cervical cancer HeLa cells (Procell Life Science & Technology Co., Ltd) were maintained in Minimum Essential Medium α (Gibco) with 10% FBS. Both 293T and HeLa cells were split every 2–3 days. Luciferase‐expressing Nalm‐6 cells, kindly provided by Professor Min Wang (Leukemia Center, Institute of Hematology and Blood Diseases Hospital, Chinese Academy of Medical Sciences, Tianjin, China), were cultured as previously described.^30^

### 2.2 Plasmid construction and retrovirus production

**FIGURE 1:**
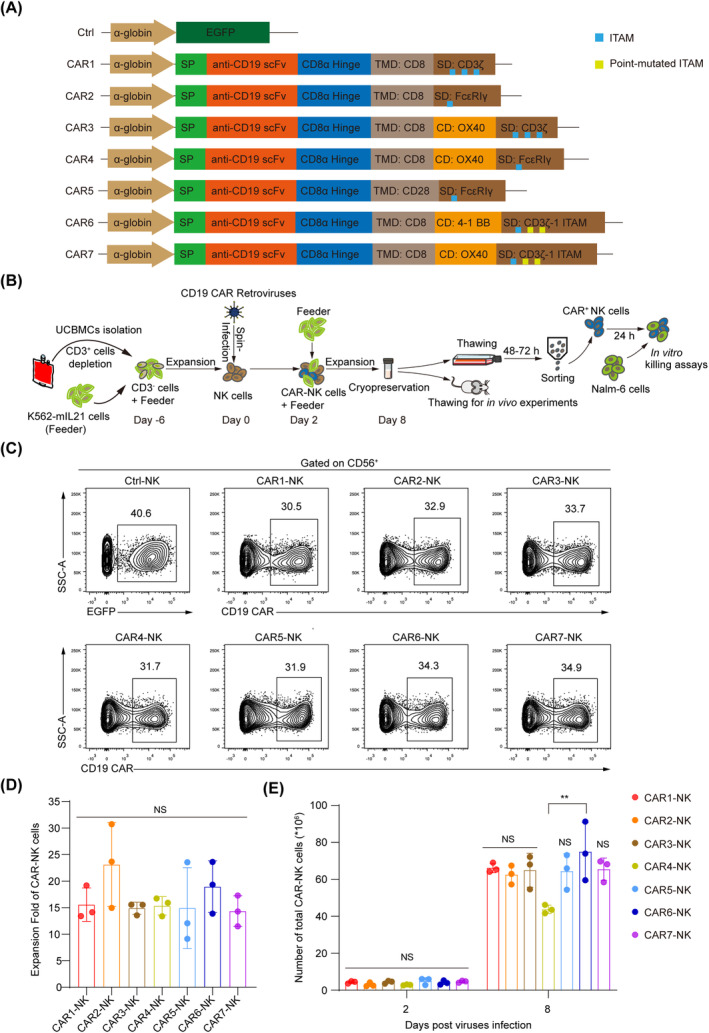
Construction and expression analysis of seven anti‐CD19 CARs. (A) Structure schematic diagrams of the expression cassettes for seven CARs targeting CD19 antigen, labelled as CAR1 to CAR7. All the CARs utilize the same scFv fragment but differ in their transmembrane and intracellular signalling domains. The control vector contains an EGFP coding sequence, denoted as Ctrl. α‐globin, promoter, SP, signal peptide, TMD, transmembrane domain, CD, co‐stimulatory domain, SD, signalling domain. (B) Schematic of CAR‐NK cell generation procedure and application scenarios. (C) Flow cytometric analysis of the infection rate of seven CD19 CAR‐NK or Ctrl‐NK cells, all retroviruses (MOI = 5) were respectively transduced into NK cells (10 million cells/group) by spin infection. The infection rates were analysed 48 h post‐transduction. Data are representative of three independent experiments. (D, E) Statistic analysis of the expansion fold (D) and cell counts (E) of the seven groups of CAR‐NK cells. Ten million NK cells were transduced with each CAR virus and expanded with K562‐mIL‐21 feeder cells for 6 days. The absolute numbers of CAR‐NK cells were analysed. Data are grouped from three independent experiments. Statistics: one‐way ANOVA and Kruskal–Wallis tests, **p < 0.01, NS, not significant. ANOVA, analysis of variance; CAR, chimeric antigen receptor; MOI, multiplicity of infection; NK, natural killer.

SFG recombinant retroviral vectors^31^ (Addgene, Plasmid #22493) were used to express designed cassettes (EGFP, seven anti‐CD19 CAR constructs). Constructs included an immunoglobulin heavy‐chain signal peptide (SP), the anti‐CD19 single‐chain variable fragment (scFv) from the FMC63 murine monoclonal antibody, the CD8α hinge domain, a TMD of CD8 or CD28, a cytoplasmic CD of OX40 or 4‐1BB and a SD of CD3ζ, CD3ζ‐1 ITAM, or FcεRIγ. The control retroviral vector was engineered by replacing the CD19 CAR constructs with the EGFP coding sequence. Detailed vector architectures are described in Figure 1A. Retroviral supernatants were produced and concentrated using Amicon Ultra‐15 Centrifugal Filters (Millipore) and stored at −80°C. Viral titres were determined using HeLa cells.^32^

### 2.3 Generation of CAR‐modified NK cells

Umbilical cord blood (UCB) units were provided by Guangdong Cord Blood Bank (Guangzhou, China). Umbilical cord blood mononuclear cells (UCBMCs) were isolated using a density‐gradient centrifugation technique (Lymphoprep, Serumwerk Bernburg AG). CD3^−^ UCBMCs, purified using CD3 biotin (BioLegend, HIT3a) and Anti‐biotin MicroBeads UltraPure kit (Miltenyi Biotec), were stimulated with K562‐mIL‐21 cells (Hangzhou Zhongying Biomedical Technology Co., Ltd) and recombinant human IL‐2 (Miltenyi Biotec, 200 U/mL) in KBM581 Serum‐free Medium (Corning) on Day −6.^33^ On Day 0, activated NK cells were transduced with retroviral supernatants at a multiplicity of infection (MOI) of 5, following the Vectofusin‐1 (Miltenyi Biotec) based transduction protocol. Infection rates were assessed by flow cytometry on Day 2. After 6 days of expansion of the CAR‐NK cells, CAR‐transduced NK cells were harvested and cryopreserved with serum‐free cell freezing medium (CELLSAVING, NCM Biotech). For animal experiments, a portion of the cryopreserved cells was thawed, washed and immediately used for tail vein injection, to mimic clinical settings of ‘off‐the‐shelf’ cell products. The absolute CAR‐NK cell numbers are equivalents of the total live NK cells multiplying CAR ratios. Another portion of cryopreserved cells was thawed, washed and cultured for 48 or 72 h. After cell recovery, the live CAR^+^ NK cells (DAPI^−^CD3^−^CD56^+^CAR^+^) were sorted using FACS for in vitro assays.

### 2.4 Flow cytometry

Transduced UCB‐NK cells were stained with fluorochrome‐conjugated antibodies against CD3 (UCHT1) and CD56 (HCD56) (all from BioLegend), and anti‐FMC63 scFv (BioSwan) for CAR expression analysis using a BD LSRFortessa flow cytometer (BD Biosciences). CD56 and anti‐FMC63 scFv were used to sort transduced UCB‐NK cells for CD56^+^CAR^+^ NK cells using Sony MA900 (SONY). Data were analysed with FlowJo software (FlowJo_v10.8.1). To assess the expression of NK receptor and effector molecules, we used antibodies against CD56, TRAIL (RIK‐2), FasL (NOK‐1), NKp30 (P30‐15), NKp44 (P44‐8), NKG2D (1D11), CD319 (162.1), CD16 (3G8), CD69 (FN50), NKG2A (S19004C), CD94 (HP‐3D9), CD96 (NK92.39), GzmB (QA18A28), Perforin (dG9), 2B4 (C1.7), DNAM‐1 (11A8), NKp46 (9E2), TIGIT (A15153G), TIM‐3 (F28‐2E2), PD‐1 (EH12.2H7) (all from BioLegend) and anti‐FMC63 scFv. Cells were resuspended in DAPI (Sigma‐Aldrich) for analysis with the BD LSRFortessa cytometer (BD Biosciences).

### 2.5 Specific cytotoxicity and serial killing assays

For cytotoxicity assessment, CAR‐transduced NK cells (CAR‐NK) or UCB‐NK cells (Effector, E) were co‐cultured with Nalm‐6 tumour cells (Target, T) labelled with eBioscience™ Cell Proliferation Dye eFluor™ 670 (eFluor 670) (Invitrogen) in U‐bottom 96‐well plates for 4 h at various E:T ratios (0:1, 0.2:1, 0.4:1, 0.8:1 and 1.6:1). Target cell death was quantified using the BD LSRFortessa flow cytometer (BD Biosciences) by measuring the percentage of DAPI in the eFluor 670‐positive population. In serial target killing assay, seven types of CD19 CAR‐NK, Mock‐NK and Ctrl‐NK cells were co‐cultured with eFlour670‐labelled Nalm‐6 cells for 12 h (Round 1) at E:T = 1:1. Fresh eFlour670‐labelled Nalm‐6 cells were added into all wells co‐cultured with the remaining effector cells for another 12 h (Round 2) at the same E:T ratio. This process was repeated for a third round (Round 3). Specific cytotoxicity was calculated using the formula: (percentage of specific death − percentage of spontaneous death) × 100.

### 2.6 CD107a expression, IFN‐γ and TNF‐α staining

Transduced UCB‐NK cells, co‐cultured with or without Nalm‐6 cells at E:T ratio of 0.5:1 for 4 h, were assessed for CD107a expression. After incubation, cells were stained with anti‐FMC63 scFv, CD56 and CD107a antibodies. For IFN‐γ and TNF‐α staining, NK cells were stimulated with Nalm‐6 cells (E:T = 0.5:1) for 2 h, then treated with BFA/Monensin (MULTISCIENCES) for an additional 2‐h incubation. Cells were stained with CD56 and anti‐FMC63 scFv antibodies, then fixed and permeabilized using the FIX & PERM Kit (MULTI SCIENCES), followed by intracellular staining with antibodies against IFN‐γ (4S.B3) or TNF‐α (MAb11) (all from BioLegend). Cells were analysed using the BD LSRFortessa cytometer (BD Biosciences).

### 2.7 Construction of B‐ALL xenograft models and NK cell treatment

NCG mice (NOD/ShiLtJGpt‐*Prkdc* ^em26Cd52^ *Il2rg* ^em26Cd22^/Gpt strain, GemPharmatech Co., Ltd.) were intravenously injected with luciferase‐expressing Nalm‐6 (Nalm‐6 luci^+^) cells (1 × 10^5^ cells/mouse) to establish human B leukaemia xenograft models on Day −1. On Day 0, tumour engraftment was quantified using bioluminescent imaging (BLI, IVIS Spectrum PerkinElmer). After irradiation (1.0 Gy, Rad Source RS2000), the animals received an intravenous injection of the equivalent of 5 × 10^6^ thawed CD19 CAR^+^ NK cells or Mock‐NK cells. Recombinant human IL‐2 (rhIL‐2, 10,000 IU/mouse, Sino Biological) was administered via intraperitoneal injection every 2 days until Day 14 post‐infusion. Tumour progression was monitored weekly by BLI. Mice with significant tumour burden were humanely euthanized. All animal experiments were performed following the guidelines of the Institutional Animal Care and Use Committee (IACUC) at the Institute of Zoology, Chinese Academy of Sciences (IOZ‐2021‐182).

### 2.8 Statistical analysis

Quantitative analysis was conducted using SPSS (IBM SPSS Statistics 25). The Shapiro–Wilk normality test assessed data distribution. Quantitative differences (mean ± SD) between samples were compared using two‐tailed Student's *t* test, Mann–Whitney *U* test, one‐way analysis of variance (ANOVA) and Kruskal–Wallis tests. *p* Values were two‐sided, with *p* < 0.05 considered statistically significant. Survival curves for tumour‐bearing mice were plotted using the Kaplan–Meier method, and differences in survival rates were compared using the Log‐rank (Mantel‐Cox) test. Statistical analyses were performed using GraphPad Prism software.

## 3 RESULTS

### 3.1 Generation of CD19 CAR‐NK cells using seven distinct CAR constructs

To enhance the efficacy of CD19 CAR‐NK cells, we engineered seven distinct CAR constructs as illustrated (CAR1–CAR7) (Figure 1A). These constructs differ in their TMDs, CDs and SDs. These designs allow for a comprehensive comparison of how these components affect CAR functionality specifically in NK cells. Each construct targets the B cell antigen CD19 using the anti‐CD19 scFv FMC63.^34^ We linked the scFv to a CD8α hinge and a CD8 or CD28 TMD, followed by an SD (CD3ζ, CD3ζ‐1 ITAM, or FcεRIγ), used alone or combined with 4‐1BB or OX40 CD^5^, ^11^, ^14^, ^35^ (Figure 1A). Notably, the CD3ζ‐1 ITAM variant includes two‐point mutations in the ITAMs, located in the second and third positions from the membrane‐proximal to the membrane‐distal direction.^14^ These CAR elements were incorporated into the SFG vector, a murine leukaemia virus‐based retroviral vector.^31^ The control vector contained an EGFP coding sequence instead of the CAR component. For retroviral particle generation, 293T cells were transfected with the SFG vector encoding the CAR cassette, a helper plasmid for GAG and Pol proteins of murine leukaemia virus and an envelope plasmid with the RD114 gene. The retroviral supernatants were harvested at 48‐ and 72‐h post‐transfection and concentrated using Amicon Ultra‐15 Centrifugal Filters. The virus titre was determined using HeLa cells.^32^

To mimic scalable and ‘off‐the‐shelf’ CAR‐NK cells for adoptive immunotherapy, we utilized cryopreserved CAR‐NK cells in our functional assays (Figure 1B). Specifically, the retroviruses (MOI = 5) were transduced into activated, T cell‐depleted (CD3^−^) umbilical cord blood‐derived NK (UCB‐NK) cells (10 million cells/group) via spin infection to prepare the CD19 CAR‐NK cells (Figure 1B). The processes for T cell depletion and NK cell activation were conducted as previously described.^22^ The purity of CD3^−^ cells after T cell depletion was 100% (Supporting Information S1: Figure S1A). The expression of CD19 CAR was assessed by flow cytometry 2 days post retroviral infection, revealing comparable infection rates of 30%–35% across the different viral groups (Figure 1C). Subsequently, the CAR‐NK cells were expanded for 6 days using K562‐mIL‐21 feeder cells. The expanded CAR‐NK cells were then counted and cryopreserved in liquid nitrogen for further functional analysis (Figure 1B, Supporting Information S1: Figure S1B). A 6‐day culture achieved an average of 14‐ to 23‐fold expansion of CAR‐NK cells after three independent experiments with different UCB donors (Figure 1D, Supporting Information S1: Table S1). Of note, the CAR6 (CD8 TMD‐4‐1BB CD‐CD3ζ 1‐ITAM SD)‐NK cells were expanded to a peak of approximately 75 million, whereas the CAR4 (CD8 TMD‐OX40 CD‐FcεRIγ SD)‐NK cells reached a minimum of approximate 44 million (CAR6 vs. CAR4, *p* < 0.01) (Figure 1E, Supporting Information S1: Table S1). Nonetheless, the total numbers of CAR‐NK cells of the other five constructs were comparable to those of expanded CAR6‐NK cells. Collectively, we successfully designed and transduced seven anti‐CD19 CAR constructs into NK cells and observed variable expansion efficiency in the presence of K562‐mIL‐21 cells.

### 3.2 Superior anti‐tumour activity of CAR1‐NK cells over other six CAR‐NK cells

**FIGURE 2:**
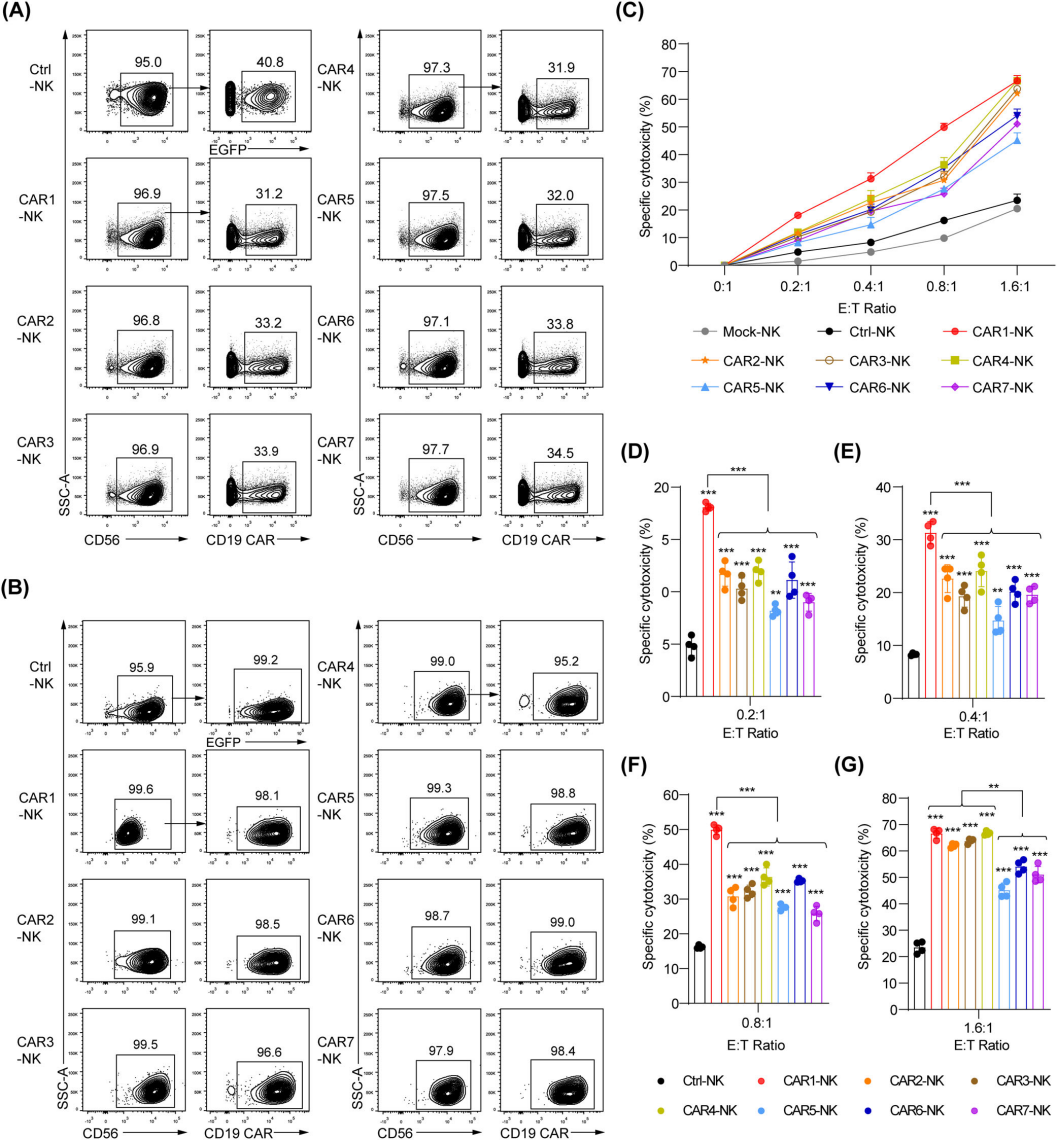
NK cell activities were enhanced by seven anti‐CD19 CAR constructs. (A) Analysis of the EGFP or CAR expression of the thawed Ctrl‐NK and CAR‐NK cells before sorting. The cryopreserved NK or CAR‐NK cells were thawed and followed by 48–72 h culture. (B) The sorting purity analysis of sorted CD3^−^CD56^+^EGFP^+^ (Ctrl‐NK) and CD3^−^CD56^+^CAR^+^ (CAR‐NK) cells post 24 h culturing. Data are representative of three independent experiments. (C) Cytotoxicity analysis of Mock‐NK, Ctrl‐NK and CAR‐NK cells (*n* = 4 per group). Seven groups of CAR‐NK cells, Mock‐NK and Ctrl‐NK cells were co‐cultured with Nalm‐6 tumour cells for 4 h at the indicated effector (E)‐to‐target (T) ratios (E:T). Specific cytotoxicity was calculated using the formula: (percentage of specific death − percentage of spontaneous death) × 100. (D–G) Analysis of specific cytotoxicity at the specific E:T ratios of 0.2:1 (D), 0.4:1 (E), 0.8:1 (F) and 1.6:1 (G). (CAR1‐NK, CAR2‐NK, CAR3‐NK, CAR4‐NK vs. CAR5‐NK, CAR6‐NK, CAR7‐NK, *p* < 0.01 at E:T = 1.6:1). Statistics: one‐way ANOVA, **p < 0.01, ***p < 0.001. ANOVA, analysis of variance; CAR, chimeric antigen receptor; NK, natural killer.

To explore the activation and persistence traits of the seven types of engineered CD19 CAR‐NK cells, we revived the cryopreserved CAR‐NK cells by incubating them for 48–72 h. Then, we sorted the FMC63^+^ (CD19 CAR^+^) NK cells and cultured them for an additional 24 h in preparation for tumour‐killing assay (Figure 2A,B). We assessed the specific cytotoxic capabilities of these CAR‐NK cells against the B cell acute lymphoblastic leukaemia (B‐ALL) line Nalm‐6. After a 4‐h co‐incubation period, all seven types of CD19 CAR‐NK cells exhibited significant cytotoxicity, outperformed that of both Mock‐NK (unmodified NK) and Ctrl‐NK (EGFP‐NK) cells (Figure 2C). Notably, CAR1 (CD8 TMD‐CD3ζ SD)‐NK cells showed the best performance in anti‐tumour activity, particularly at effector‐to‐target (E:T) ratios of 0.2:1, 0.4:1 and 0.8:1 (CAR1‐NK vs. other six CAR‐NK, *p* < 0.001) (Figure 2D–G, Supporting Information S1: Table S2). While the differences in specific cytotoxicity between CAR1‐NK cells and the other CAR‐NK cells variants decreased, CAR1‐NK cells consistently maintained superior anti‐tumour activity at the E:T ratio of 1.6:1 (Figure 2G). At higher E:T ratios, CAR1‐, CAR2‐, CAR3‐ and CAR4‐NK cells formed a subgroup with no obvious differences in performance (E:T = 1.6:1, CAR1‐NK, CAR2‐NK, CAR3‐NK, CAR4‐NK vs. CAR5‐NK, CAR6‐NK, CAR7‐NK, *p* < 0.01) (Figure 2G). However, CAR5 (CD28 TMD‐FcεRIγ SD)‐, CAR6‐ and CAR7 (CD8 TMD‐OX40 CD‐CD3ζ 1‐ITAM SD)‐NK cells formed a subgroup and exhibited comparatively lower cytotoxicity. Among these, CAR5‐NK cells showed the weakest specific cytotoxicity against Nalm‐6 cells (Figure 2D–G, Supporting Information S1: Table S3).

Taken together, these findings suggest that the CAR1‐NK cells outperform the other six types of CAR‐NK cells at lower E:T ratios. At higher E:T ratios, CAR1‐NK cells, along with CAR2‐, CAR3‐ and CAR4‐NK cells, show similar cytotoxicity. However, CAR5‐, CAR6‐ and CAR7‐NK cells exhibit weaker cytotoxicity at varying E:T ratios.

### 3.3 CAR1‐NK cells exhibited elevated CD107a expression, along with comparable levels of NK cell receptors and effectors in comparison to other CAR‐NK cells

**FIGURE 3:**
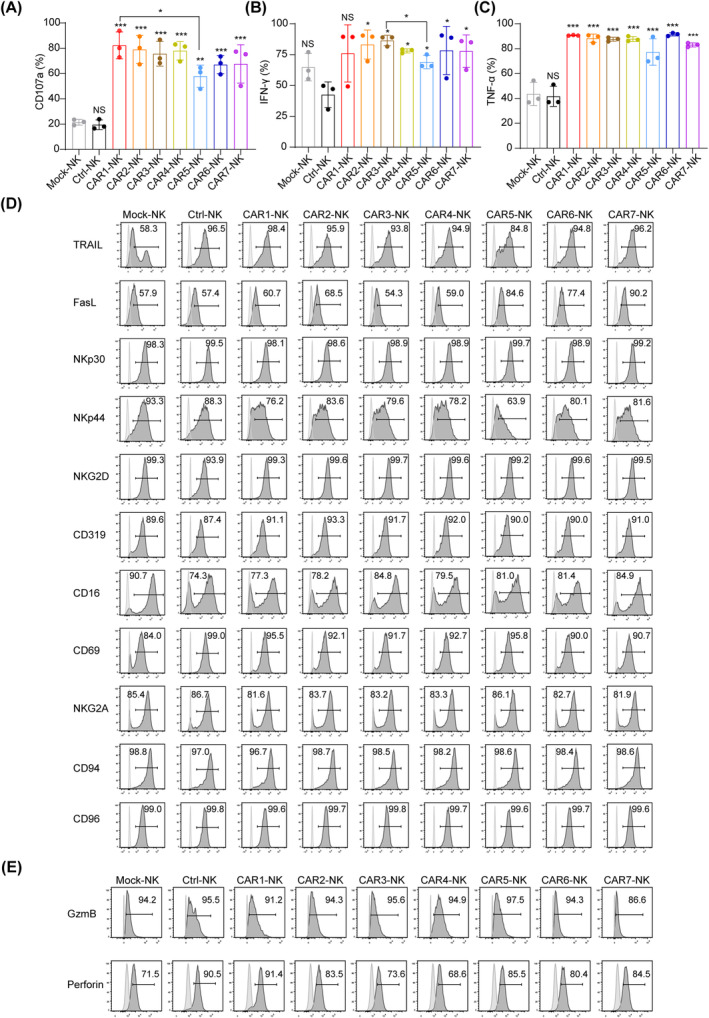
Molecular characterization of seven CD19 CAR‐NK cells. (A) Measurement of CD107a expression in CAR‐NK cells in response to Nalm‐6 cells. Mock‐NK, Ctrl‐NK and CAR‐NK cells were stimulated at the E:T ratio of 0.5:1 for 4 h. CD107a^+^ cells were gated on CD56^+^ (Mock‐NK), CD56^+^EGFP^+^ (Ctrl‐NK) or CD56^+^CAR^+^ (CAR‐NK) cells. Statistics: one‐way ANOVA and two‐tailed Student's *t* test, Mock‐NK versus other NK groups, ***p < 0.001, **p < 0.01, *p < 0.05, NS, not significant. (B, C) Assessment of IFN‐γ (B) and TNF‐α (C) production by Mock‐NK, Ctrl‐NK and CAR‐NK cells following 4‐h co‐culture with Nalm‐6 tumour cells. IFN‐γ^+^ and TNF‐α^+^ cells were gated on CD56^+^ (Mock‐NK), CD56^+^EGFP^+^ (Ctrl‐NK) and CD56^+^CAR^+^ (CAR‐NK) cells. Statistics: one‐way ANOVA, two‐tailed Student's *t* test, and Mann–Whitney *U* test, Mock‐NK or Ctrl‐NK versus other NK groups, ***p < 0.001, *p < 0.05, NS, not significant. (D, E) The expression analysis of apoptosis‐related ligands (TRAIL and FasL), typical NK cell receptors (NKp30, NKp44, NKG2D, CD319, CD16, CD69, NKG2A, CD94 and CD96) (D), and effector molecules (GzmB and Perforin) (E). Control histograms represent FMO control. CAR, chimeric antigen receptor; IFN‐γ, interferon‐gamma; NK, natural killer; TNF‐α, tumour necrosis factor‐alpha.

To explore the molecular characteristics of seven CD19 CAR‐NK cells at the protein level, we performed the NK cell stimulation assay by co‐culturing the CAR‐NK cells with Nalm‐6 cells at the E:T ratio of 0.5:1 for 4 h. We then analysed CD107a, a membrane protein associated with NK cell cytotoxic activity,^36^ along with interferon‐gamma (IFN‐γ) and tumour necrosis factor‐alpha (TNF‐α), which are also related to NK cell cytotoxicity.^37^ Our results showed that all seven CAR‐NK cells exhibited higher levels of CD107a expression compared to Mock‐NK and Ctrl‐NK cells (Figure 3A). Notably, CAR1‐NK cells exhibited the highest CD107a expression level, aligning with their superior in vitro cytotoxicity (Figure 2C–G). CAR2‐, CAR3‐ and CAR4‐NK cells showed similar CD107a expression levels, which were higher than those observed in CAR5‐, CAR6‐ and CAR7‐NK cells (Figure 3A). Moreover, all CAR‐NK cells produced IFN‐γ and TNF‐α upon stimulation by Nalm‐6 cells. Interestingly, the expression level of these two cytokines did not correlate with the cytotoxic capabilities of CAR‐NK cells in vitro (Figure 3B,C).

We also measured the expression of NK cell surface receptors on the CAR‐NK cells. Indeed, all seven types of CAR‐NK cells highly expressed classical NK cell surface receptors, including apoptosis‐related ligands (TRAIL and FasL), activating receptors (NKp30, NKp44, NKG2D, CD319, CD16 and CD69) and inhibitory receptors (NKG2A, CD94 and CD96)^38^ (Figure 3D). Phenotypic analysis further revealed that all seven CD19 CAR‐NK cells exhibited significant expression of cytotoxic granules, such as granzyme B (GzmB) and perforin (Figure 3E). Collectively, these molecular features indicate that all seven types of CD19 CAR cells display classical NK cell hallmarks. The expression levels of CD107a particularly highlight the effectiveness of CAR construct designs in the context of NK cells.

### 3.4 Robust and comparable serial killing abilities of seven CD19 CAR‐NK cells in vitro

**FIGURE 4:**
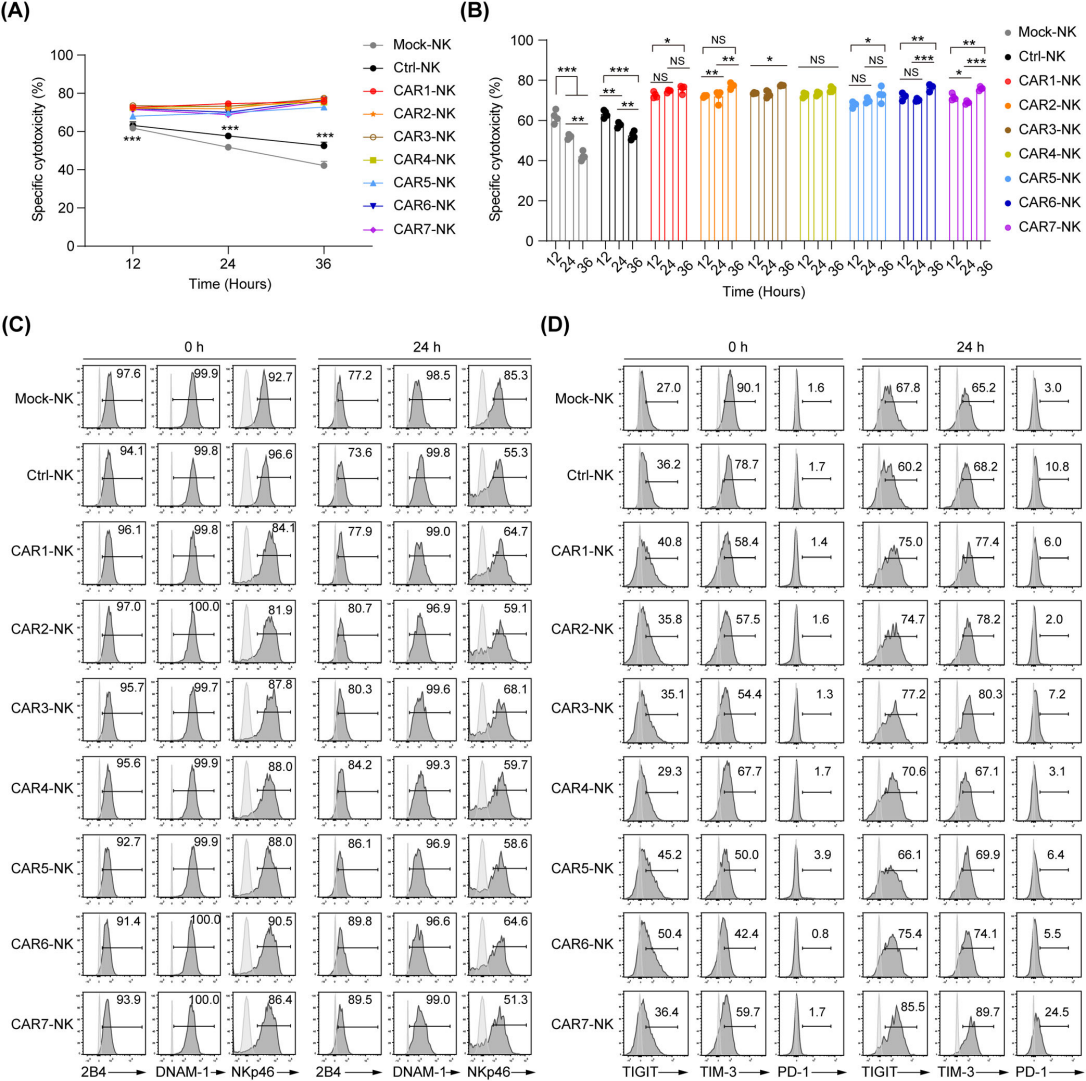
Sustained cytotoxicity and molecular features in seven CD19 CAR‐NK cells across multiple rounds of killing. (A, B) Cytotoxicity analysis of three consecutive rounds of Nalm‐6 cell killing of the CAR‐NK cells (*n* = 4 per group). Mock‐NK, Ctrl‐NK and seven groups of CAR‐NK cells were respectively co‐cultured with Nalm‐6 cells for 12 h per round at the E:T ratio of 1:1. The fresh Nalm‐6 cells were added to the NK cell residues incubated every other 12 h. Specific cytotoxicity was calculated using the formula: (percentage of specific death − percentage of spontaneous death) × 100. Statistics: one‐way ANOVA and Kruskal–Wallis tests, ***p < 0.001, **p < 0.01, *p < 0.05, NS, not significant. Round 1, round 2 and round 3, Ctrl‐NK versus CAR‐NK, *p* < 0.001. (C, D) Flow cytometric analysis of NK cell activation (2B4, DNAM‐1 and NKp46) (C) and exhaustion markers (TIGIT, TIM‐3 and PD‐1) (D) before and after two rounds of killing. Unstained control is indicated in open histograms. ANOVA, analysis of variance; CAR, chimeric antigen receptor; NK, natural killer.

To evaluate the persistent cytotoxic activity of seven CD19 CAR‐NK cells in serial killing assays, we conducted three rounds of tumour‐killing assays^39^ with Nalm‐6 tumour cells (E:T = 1:1). Initially, after a 12‐h incubation (Round 1), fresh Nalm‐6 cells were added to the co‐culture wells containing residual NK or CAR‐NK cells for another 12 h (Round 2). Following this, the final round of Nalm‐6 cells was added to the co‐culture wells for another 12 h (Round 3). Throughout these three rounds of tumour‐killing, all seven types of CAR‐NK cells consistently exhibited robust cytotoxicity. This serial killing feature of CAR‐NK cells was not seen in Mock‐NK or Ctrl‐NK cells, which demonstrate diminishing tumour‐killing abilities (Figure 4A,B).

In addition, we analysed the classical activation effectors (2B4, DNAM‐1 and NKp46) and exhaustion markers (TIGIT, TIM‐3 and PD‐1) in all types of CAR‐NK cells before incubation and after two rounds of target killing.^40^, ^41^ The data revealed that each group maintained similar expression levels of activation and exhaustion receptors, preserving these markers at stable levels even after repeated exposure to Nalm‐6 cells, coupled with a general increase in exhaustion marker expression (Figure 4C,D). Interestingly, CAR2‐NK and CAR4‐NK cells exhibited lower expression levels of the exhaustion receptor PD‐1, while CAR7‐NK cells showed the highest level. These findings suggest that the FcεRIγ SD may mitigate exhaustion in CAR‐NK cells, and that the CAR7 construct might induce greater exhaustion in NK cells during targeted tumour killing. Overall, these results demonstrate that CAR‐NK cells maintain robust cytotoxicity and typical molecular features of activation and exhaustion status across multiple rounds of tumour‐killing assays.

### 3.5 Enhanced anti‐tumour efficacy of CAR1‐, CAR2‐, CAR3‐ and CAR4‐NK cells in xenograft animals

**FIGURE 5:**
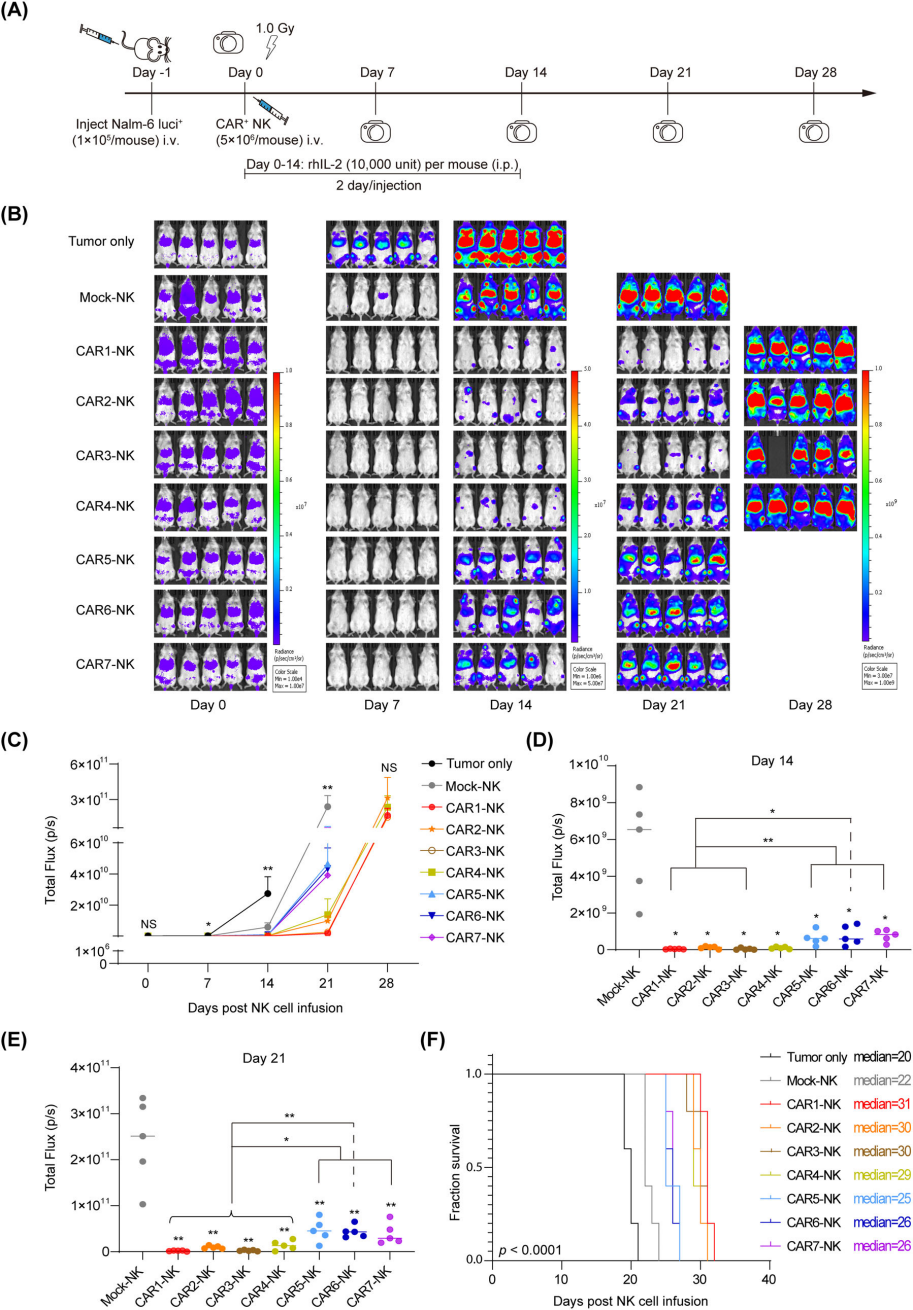
Inhibition of human B leukaemia development in xenograft models by seven types of CD19 CAR‐NK cells. (A) Schematic diagram of in vivo studies with luciferase‐expressing (luci^+^) Nalm‐6 cells (1 × 10^5^ cells/mouse, i.v.) in mouse xenograft models. The equivalent of 5 million CD19 CAR‐NK cells were infused into each animal. rhIL‐2 (10,000 IU/mouse, i.p.) was administered every 2 days until Day 14 post‐NK infusion. (B) Bioluminescence imaging (BLI) of xenograft models (Tumour only, Tumour + Mock‐NK, and Tumour + seven CD19 CAR‐NK, *n* = 5 animals per group). (C) Statistics of the total flux (p/s) of each group after NK cell infusion. Statistics: Kruskal–Wallis tests, one‐way ANOVA, and two‐tailed Student's *t* test. On Day 7, Tumour only versus Mock‐NK, *p* < 0.05. On Day 14, Tumour only versus Mock‐NK, *p* < 0.01. On Day 21, Mock‐NK versus seven CAR‐NK, *p* < 0.01. **p < 0.01, *p < 0.05, NS, not significant. (D, E) BLI quantification of tumour burden on Day 14 and 21, displayed as mean ± SD. Mock‐NK versus each CAR‐NK group; statistics: one‐way ANOVA and two‐tailed Student's *t* test. **p < 0.01, *p < 0.05. (F) Kaplan–Meier survival curves of the xenograft models. Comparisons between groups provided with specified *p* values. Tumour only versus Mock‐NK, *p* < 0.01. Mock‐NK versus seven CAR‐NK, *p* < 0.01; statistics: two‐tailed log‐rank test. ANOVA, analysis of variance; CAR, chimeric antigen receptor; NK, natural killer.

To determine the in vivo therapeutic potential of the seven CD19 CAR‐NK cells, we established the B‐ALL xenograft animal models using the luciferase‐expressing Nalm‐6 cells (Nalm‐6 luci^+^) in the NCG (NOD/ShiLtJGpt‐*Prkdc* ^em26Cd52^ *Il2rg* ^em26Cd22^/Gpt strain) immune‐deficient mice. One day prior to treatment (Day −1), the mice were injected with 1 × 10^5^ Nalm‐6 luci^+^ cells via tail vein. These tumour‐bearing mice were then randomly divided into nine groups based on total flux values analysed by BLI on Day 0. After randomization, the mice received 1.0 Gy irradiation and were subsequently injected with the equivalent of 5 million CD19 CAR‐NK cells or Mock‐NK cells via tail vein (Supporting Information S1: Table S4). Additionally, to enhance the maintenance of CAR‐NK cells, rhIL‐2 (10,000 IU/mouse) was administered every 2 days for 2 weeks. Tumour progression in both treated and Tumour only groups was monitored by BLI every 7 days (Figure 5A). Compared to untreated tumour‐bearing mice, Mock‐NK cells exhibited transient tumour resistance, but the tumour relapsed quickly within 7‐day post‐treatment. Notably, all seven types of CD19 CAR‐NK cells significantly reduced the tumour burden for a prolonged 21‐day period post‐treatment in the treated tumour‐bearing mice (On Day 14, Tumour only vs. Mock‐NK, *p* < 0.01, Mock‐NK vs. seven CAR‐NK, *p* < 0.05. On Day 21, Mock‐NK vs. seven CAR‐NK, *p* < 0.01). Moreover, CAR1‐NK and CAR3‐NK cells demonstrated superior tumour‐eradicating efficacy compared to CAR5‐, CAR6‐ and CAR7‐NK cells (On Day 14, CAR1‐NK, CAR3‐NK vs. CAR6‐NK, *p* < 0.05, CAR1‐NK, CAR3‐NK vs. CAR5‐NK, CAR7‐NK, *p* < 0.01. On Day 21, CAR1‐NK, CAR2‐NK, CAR3‐NK, CAR4‐NK vs. CAR6‐NK, *p* < 0.01, CAR1‐NK, CAR2‐NK, CAR3‐NK, CAR4‐NK vs. CAR5‐NK, CAR7‐NK, *p* < 0.05) (Figure 5B–E). All seven types of CAR‐NK cells significantly prolonged the survival of treated tumour‐bearing mice when compared with those treated by Mock‐NK cells. Of note, animals treated with CAR1‐, CAR2‐, CAR3‐ and CAR4‐NK cells showed longer survivals and formed a dominant subgroup when compared to those treated with CAR5‐, CAR6‐ and CAR7‐NK cells (CAR1‐NK, CAR2‐NK, CAR3‐NK, CAR4‐NK vs. CAR5‐NK, CAR6‐NK, CAR7‐NK, *p* < 0.01) (Figure 5F). These in vivo findings align with our previous cytotoxicity assay results.

**FIGURE 6:**
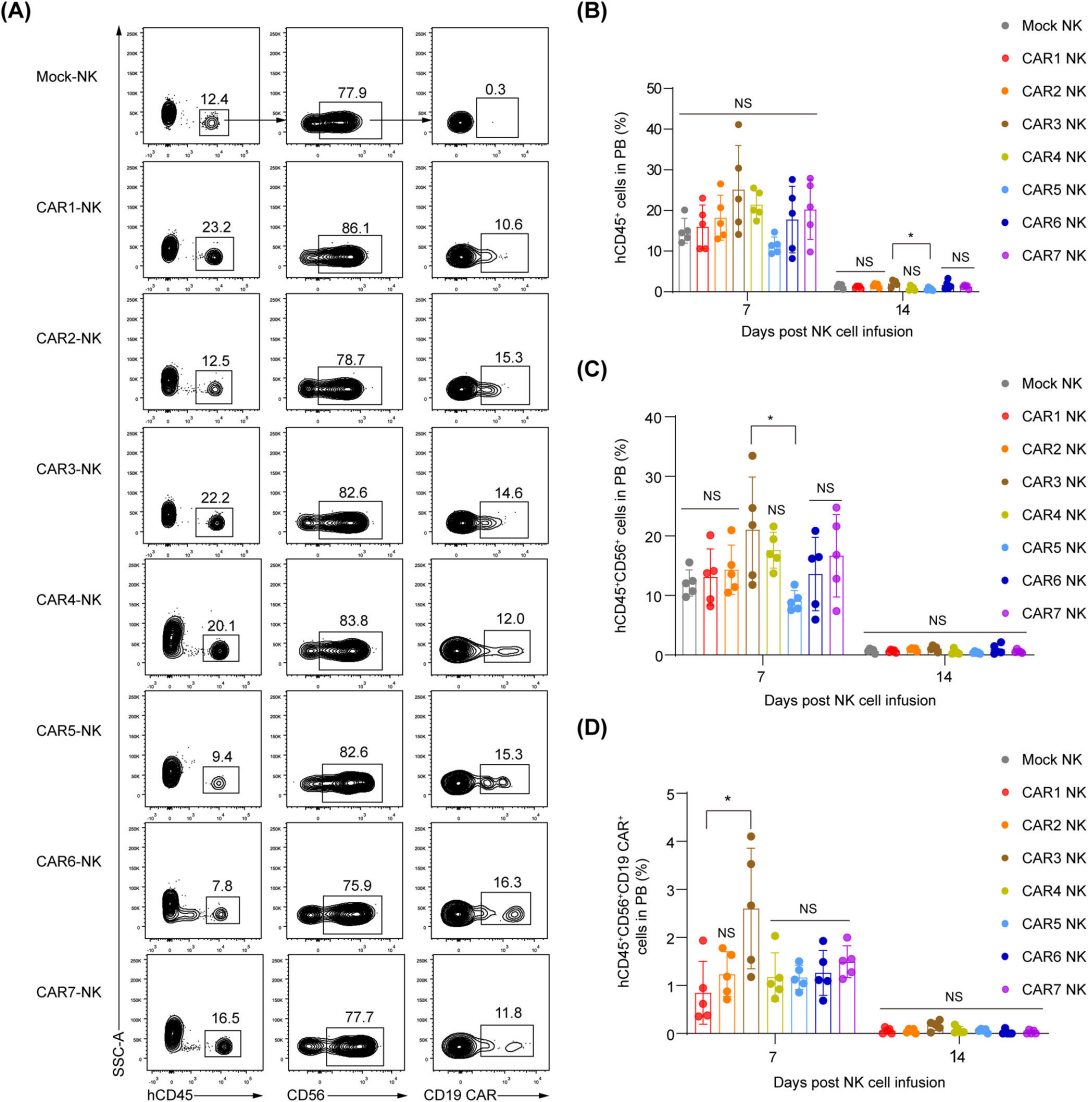
Kinetics and persistence of seven CD19 CAR‐NK cells in vivo. (A). Flow cytometric analysis of the human CD45^+^CD56^+^CD19 CAR^+^ cells in the peripheral blood (PB) of tumour‐bearing mice (*n* = 5 animals per group) on Day 7 post‐NK cell injection. Results are representative for each group. (B–D) The proportion analysis of the hCD45^+^ cell population (B), hCD45^+^CD56^+^ NK cell population (C), and hCD45^+^CD56^+^CD19 CAR^+^ cell population (D) in the PB of tumour‐bearing mice on Day 7 and 14 post‐CAR‐NK cell injection. Each dot represents an individual mouse, with median ± SD shown. Statistics: one‐way ANOVA and Kruskal–Wallis tests, CAR3‐NK versus other NK groups, *p < 0.05, NS, not significant. ANOVA, analysis of variance; CAR, chimeric antigen receptor; NK, natural killer.

Additionally, we investigated the kinetics and persistence of total human NK cells and CD19 CAR^+^ cells in each group of tumour‐bearing mice post‐CAR‐NK cell infusion. On Day 7, a detectable number of human CD45^+^CD56^+^ NK cells and CD45^+^CD56^+^CD19 CAR^+^ cells were present in the circulation of each group, as confirmed by flow cytometry (Figure 6A). However, by Day 14, the levels of human CD45^+^ cells in peripheral blood (PB) substantially decreased. Notably, the highest percentage of CAR‐NK cells was observed in the PB of mice treated with CAR3‐NK cells, while the CAR5‐NK cells exhibited the lowest level (Figure 6B–D). This observation correlates with the respective stronger or weaker tumour‐killing efficacy of CAR3‐NK and CAR5‐NK cells. Collectively, these studies demonstrate that all seven types of CD19 CAR‐NK cells mediate enhanced anti‐tumour activity, particularly when CAR constructs contain CD8 TMD and CD3ζ SD or are combined with OX40 CD.

## 4 DISCUSSION

In this study, we engineered seven CAR constructs with distinct TMDs, CDs and SDs. Our goal was to identify the most effective CAR construct for optimal anti‐tumour activity in NK cells. Except for the CAR4‐NK cells showing a weaker expansion capacity, all other six CAR‐NK cells exhibited similar expansion abilities. Moreover, seven CD19 CAR‐NK cells displayed similar phenotypes with unmodified UCB‐NK cells and exhibited significantly enhanced cytotoxicity against CD19 antigen‐expressing leukaemia cells. Notably, NK cells expressing CAR1 (CD8 TM‐CD3ζ SD), CAR2 (CD8 TM‐FcεRIγ SD), CAR3 (CD8 TM‐OX40 CD‐CD3ζ SD) and CAR4 (CD8 TM‐OX40 CD‐FcεRIγ SD) demonstrated effective anti‐tumour activity and prolonged survival in Nalm‐6 tumour xenograft animals, with CAR1‐NK cells showing the highest efficacy. Remarkably, CAR3‐NK cells significantly improved in vivo persistence in tumour‐bearing animals. This study offers valuable insights into the engineering of CAR‐NK cells for enhanced efficacy in tumour treatment.

The ITAM is the functional motif for the SD of CAR constructs, which initiates the signalling cascades and produces the effects of cell activation and expansion. CD3ζ (three ITAMs) and FcεRIγ (one ITAM) are prominent SDs extensively utilized in CAR constructs in T cells.^42^ Moreover, both CD3ζ and FcεRIγ are critical for NK cell activation and effector functions.^43^ The numbers and positions of ITAM in SD have vital roles in CAR‐T cell functionalities. Attenuating CD3ζ signalling by silencing two ITAMs via mutating tyrosine‐encoding sites within the CD3ζ domain can enhance the in vivo tumour elimination ability of CAR‐T cells by extending their functional persistence without compromising their potency.^14^ However, our results showed that CAR1‐NK cells exhibited superior anti‐tumour activity over the CAR with two ITAM mutants in NK cells. Our data indicated that attenuating the CD3ζ signalling impedes the anti‐tumour function of CAR‐NK cells, which may be due to the shorter lifespan of NK cells compared with T cells in vivo.

CAR3 containing OX40 CD exhibited superior efficacy and persistence in CAR‐NK cell‐treated tumour‐bearing animals. This finding aligns with previous studies that have shown incorporating OX40 CD into CAR constructs enhances persistence and reduces activation‐induced cell death in CAR‐T cells.^44^, ^45^ Thus, a combination of OX40 CD and CD3ζ SD might result in significant clinical potential for CAR‐NK cell therapy, as evidenced by their inclusions in clinical trials by leading biopharmaceutical companies (NCT05020678, NCT04623944). Our data revealed that CAR5 (CD28 TMD‐FcεRIγ SD) with one ITAM demonstrated the weakest activity against tumour cells. Recent studies have shown that linking CARs with CD28 hinge and TMD, rather than CD8 hinge and TMD, increased risks of cytokine release syndrome and neurotoxicity, and impaired persistence of CAR‐T cells.^46^, ^47^ This may explain the poor efficacy observed in our CAR5‐NK cells. Additionally, the expression levels and intensities of CAR on seven CAR‐NK cells in the PB of tumour‐bearing mice were lower than those in vitro, which was mainly due to the degradation and downregulation of CAR after engaging tumour antigens as reported.^48^, ^49^, ^50^

Elevated expression of exhaustion‐related molecules, including TIGIT, TIM‐3 and PD‐1, was observed in all CAR‐NK populations following prolonged target engagement, particularly in CAR7‐NK cells. As immune checkpoint receptors, TIGIT, TIM‐3 and PD‐1 correlate with the exhaustion status of NK cells.^40^, ^51^ Combining CAR‐NK cells with immune checkpoint inhibitors targeting these molecules could potentially enhance therapeutic outcomes in clinical settings.^52^

The comparison of seven CAR designs was conducted using the UCB‐NK cells and RD114‐pseudotyped retroviral expression system, which were applied in clinical trial.^22^ To reduce the variations of heterogeneity of NK cells from different genetic backgrounds, we chose the same donor‐derived UCB‐NK cells to manufacture the CAR‐NK cells for all experiments, and three donor‐derived NK cells were tested for the seven CAR constructs. Moreover, the seven packaged CAR retroviruses were adjusted to the same titre (1 × 10^8^ TU/ml) before infecting NK cells and we transduced the NK cells with the same MOI (MOI = 5) to minimize the transduction efficiency variations.

In conclusion, our comparative analysis of seven anti‐CD19 CAR designs using UCB‐derived NK cells indicates that the first‐generation CAR construct with CD8 TMD‐CD3ζ SD offers optimal anti‐tumour efficacy. For the in vivo persistence of CAR‐NK cells, the CAR3 construct containing CD8 TMD‐OX40 CD‐CD3ζ SD emerges as a superior choice. These findings provide valuable insights for the development of CAR‐NK cell products for clinical trials.

## AUTHOR CONTRIBUTIONS

Yao Wang designed and conducted all experiments, performed data analysis and wrote the manuscript. Jianhuan Li conducted all experiments and performed data analysis. Zhiqian Wang, Yanhong Liu, Fan Zhang, Dehao Huang, Lijuan Liu, Yanping Zhu, Hanmeng Qi, Leqiang Zhang and Yaoqin Zhao participated in multiple experiments. Tongjie Wang, Mengyun Zhang, Chengxiang Xia, Wenbin Qian and Xiaofan Zhu discussed the data and manuscript. Fangxiao Hu designed the project, guided all experiments, scrutinized all experimental data and wrote the manuscript. Jinyong Wang designed the project, wrote the manuscript and provided the final approval of the manuscript.

## CONFLICT OF INTEREST STATEMENT

The authors declare no conflicts of interest.

## Supporting information


**Data S1:** Supporting Information.

## Data Availability

The data that support the findings of this study are available from the corresponding author upon reasonable request.
