# Correction to “Comparison of Seven CD19 CAR Designs in Engineering NK Cells for Enhancing Anti‐Tumour Activity”

**DOI:** 10.1111/cpr.70098

**Published:** 2025-07-21

Y. Wang, J. H. Li, Z. Q. Wang, J. Li, Z. Wang, Y. Liu, T. Wang, M. Zhang, C. Xia, F. Zhang, D. Huang, L. Zhang, Y. Zhao, L. Liu, Y. Zhu, H. Qi, X. Zhu, W. Qian, F. Hu and J. Wang, “Comparison of Seven CD19 CAR Designs in Engineering NK Cells for Enhancing Anti‐Tumour Activity,” *Cell Proliferation* 57, no. 11 (2024): e13683. https://doi.org/10.1111/cpr.13683.

In Figure 1A, the promoters of the SFG vector in structure schematic diagrams were mistakenly labelled as “α‐globin”. The errors do not affect the rest of the article. The incorrect version is shown below:

**FIGURE 1** Construction and expression analysis of seven anti‐CD19 CARs. (A) Structure schematic diagrams of the expression cassettes for seven CARs targeting CD19 antigen, labelled as CAR1 to CAR7. All the CARs utilize the same scFv fragment but differ in their transmembrane and intracellular signaling domains. The control vector contains an EGFP coding sequence, denoted as Ctrl. α‐globin, promoter, SP, signal peptide, TMD, transmembrane domain, CD, co‐stimulatory domain, SD, signaling domain. (B) Schematic of CAR‐NK cell generation procedure and application scenarios. (C) Flow cytometric analysis of the infection rate of seven CD19 CAR‐NK or Ctrl‐NK cells, all retroviruses (MOI = 5) were respectively transduced into NK cells (10 million cells/group) by spin infection. The infection rates were analyzed 48 h post‐transduction. Data are representative of three independent experiments. (D, E) Statistic analysis of the expansion fold (D) and cell counts (E) of the seven groups of CAR‐NK cells. Ten million NK cells were transduced with each CAR virus and expanded with K562‐mIL‐21 feeder cells for 6 days. The absolute numbers of CAR‐NK cells were analyzed. Data are grouped from three independent experiments. Statistics: One‐way ANOVA and Kruskal‐Wallis tests, **p < 0.01, NS, not significant.

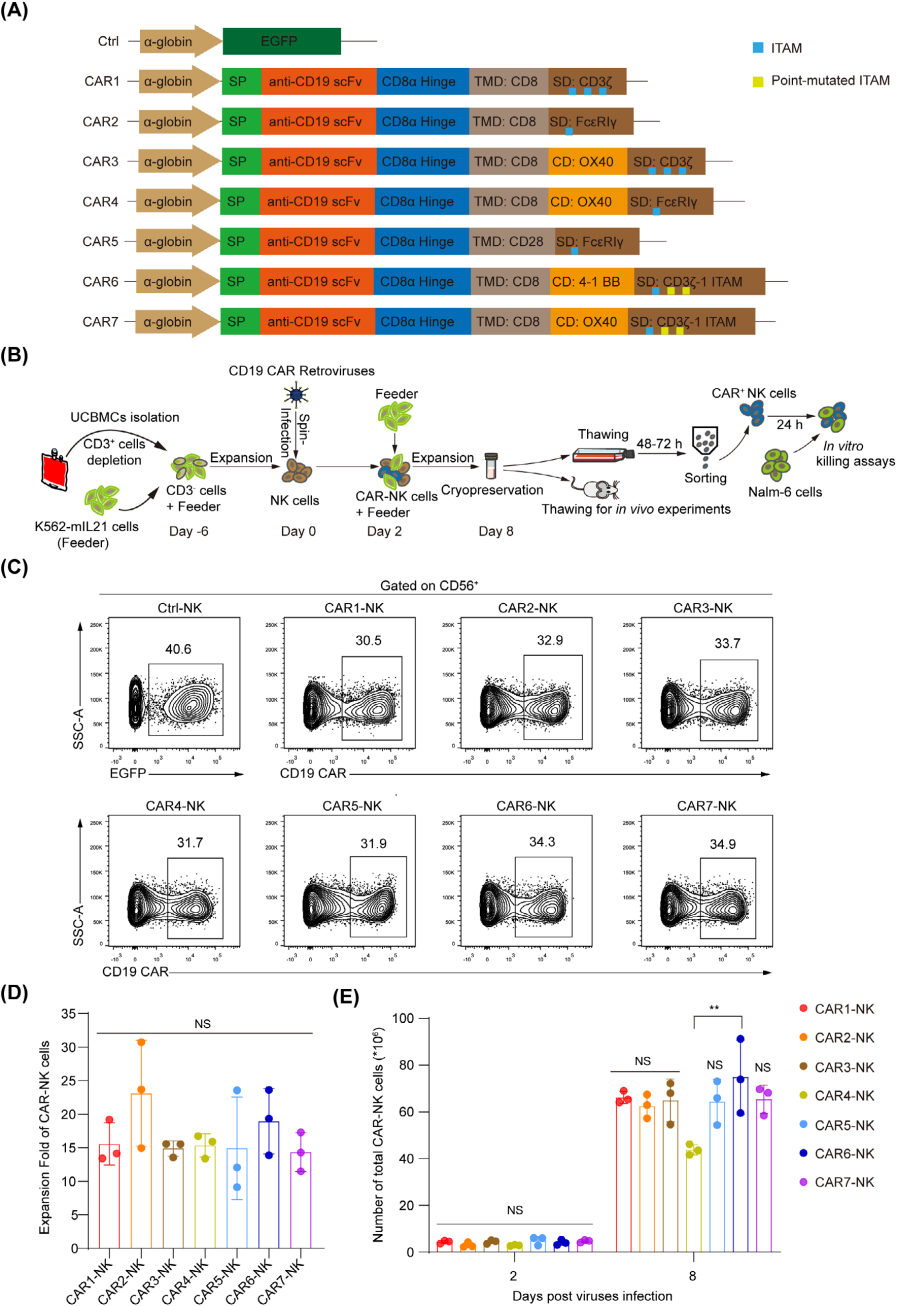

The corrected Figure 1 and accompanying legend appear below.

**FIGURE 1** Construction and expression analysis of seven anti‐CD19 CARs. (A) Structure schematic diagrams of seven CD19 CAR vectors, labelled as CAR1 to CAR7. All the CARs utilise the same scFv fragment but differ in their transmembrane and intracellular signalling domains. The control vector contains an EGFP coding sequence, denoted as Ctrl. MMLV, moloney murine leukaemia virus, LTR, long terminal repeat, ψ+, packaging signal, SA, splice acceptor, SP, signal peptide, TMD, transmembrane domain, CD, co‐stimulatory domain, SD, signalling domain. (B) Schematic of CAR‐NK cell generation procedure and application scenarios. (C) Flow cytometric analysis of the infection rate of seven CD19 CAR‐NK or Ctrl‐NK cells, all retroviruses (MOI = 5) were respectively transduced into NK cells (10 million cells/group) by spin infection. The infection rates were analysed 48 h post‐transduction. Data are representative of three independent experiments. (D, E) Statistic analysis of the expansion fold (D) and cell counts (E) of the seven groups of CAR‐NK cells. Ten million NK cells were transduced with each CAR virus and expanded with K562‐mIL‐21 feeder cells for 6 days. The absolute numbers of CAR‐NK cells were analysed. Data are grouped from three independent experiments. Statistics: One‐way ANOVA and Kruskal‐Wallis tests, **p < 0.01, NS, not significant. ANOVA, analysis of variance; CAR, chimeric antigen receptor; MOI, multiplicity of infection; NK, natural killer.

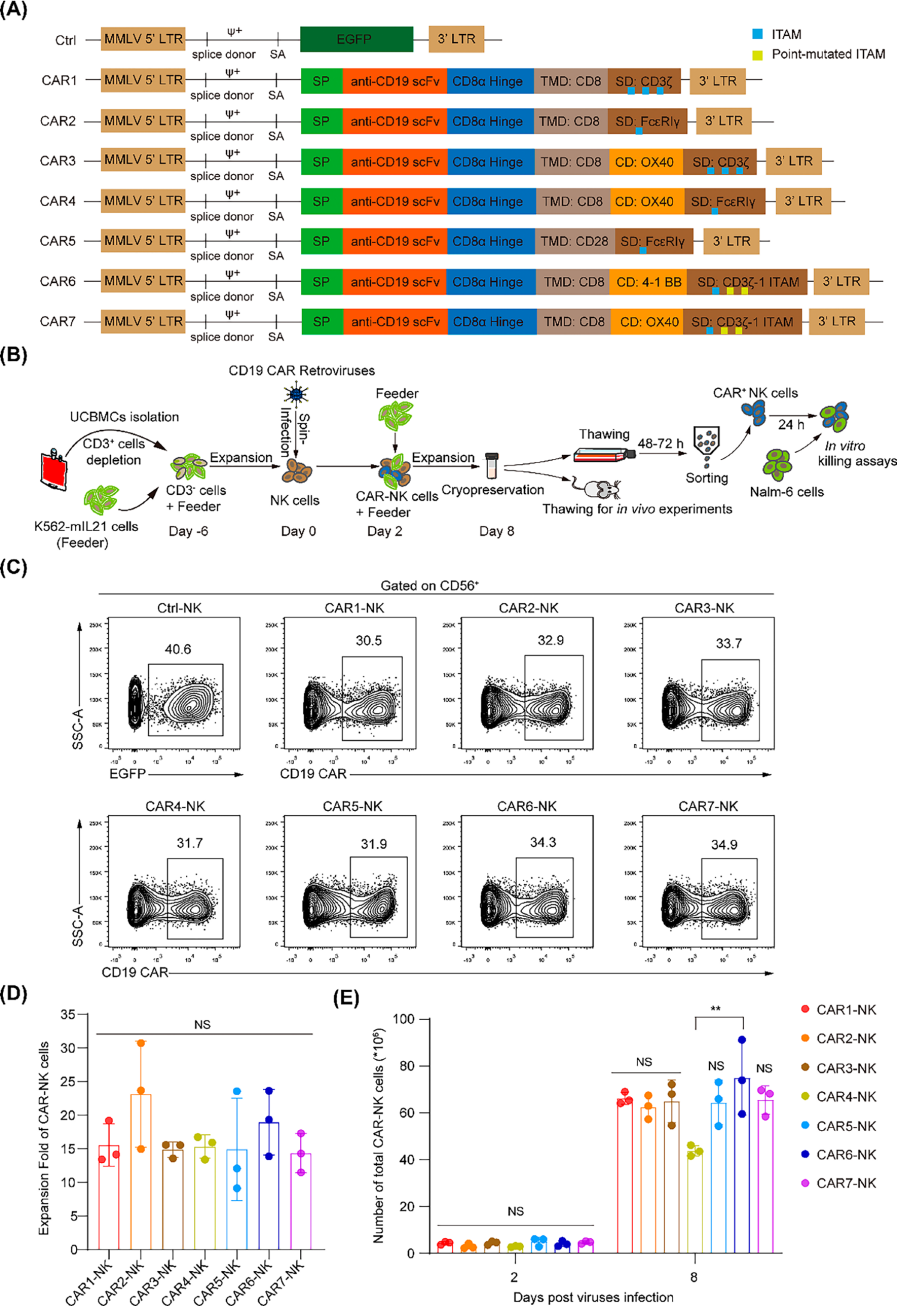

The incorrect Figure:

Corrected Figure:

We apologize for the errors.

