# Supplementary material for: Comparison of seven CD19 CAR designs in engineering NK cells for enhancing anti‐tumour activity
Source: Cell Prolif. 2024 Jun 3;57(11):e13683. doi: 10.1111/cpr.13683 (PMC11533075; doi:10.1111/cpr.13683)
Supplement: Supplementary file 1 — Data S1: Supporting Information. [file CPR-57-e13683-s001.docx]

**Supplemental Figures**

**
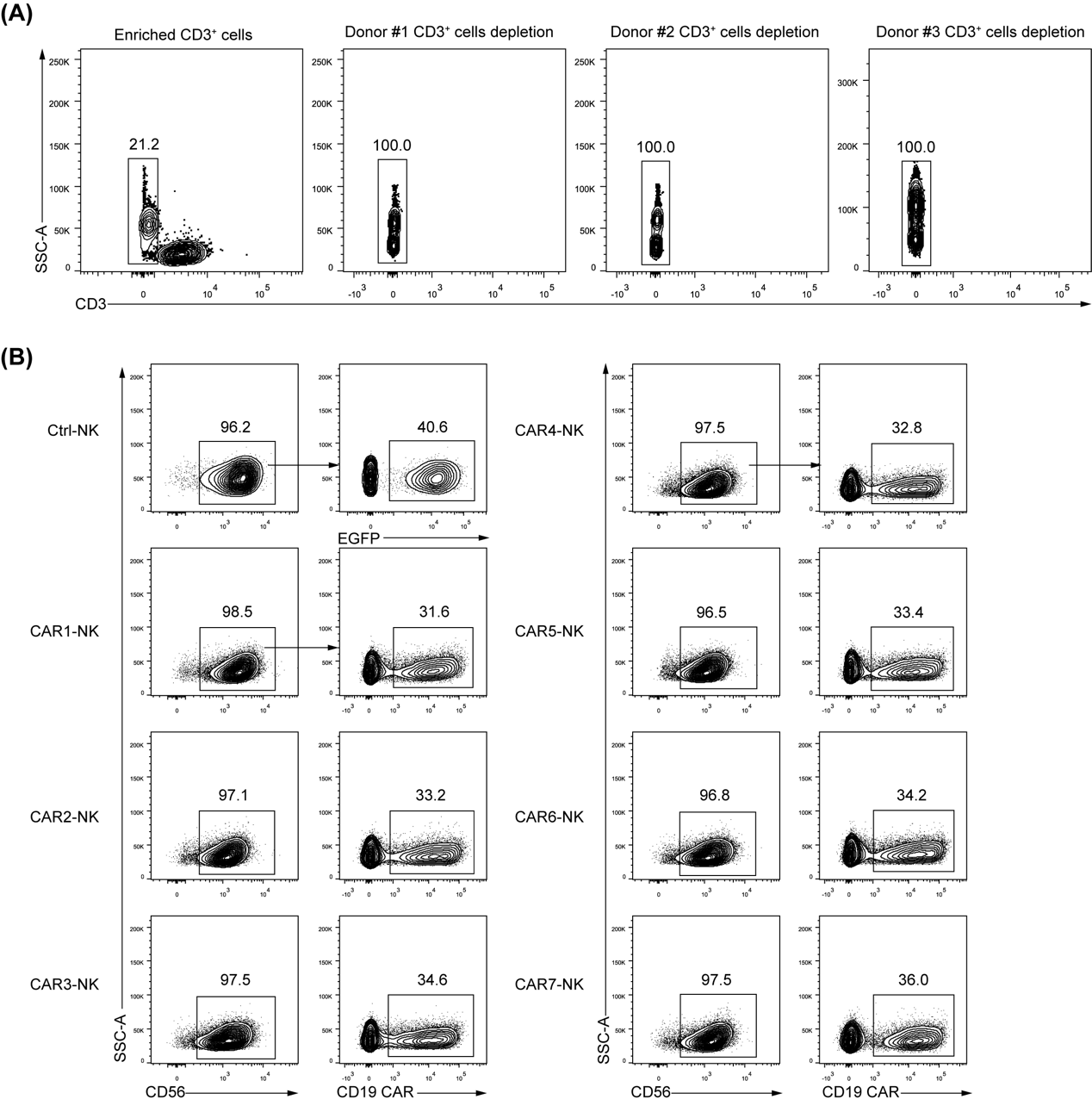
**

**Supplemental Fig.1** Quality control analysis and expansion of NK cells. (**A)** Flow cytometric analysis of the T cells (CD3^+^) residual in UCBMCs after CD3^+^ cells depletion on day -6 of three independent donors. (**B)** Analysis of the EGFP or CAR expression of Ctrl-NK and CAR-NK cells on day 8 before cryopreservation.

**Table S1.** Number of total CAR^+^ NK cells with NK cells derived from three donors.

| Group | Day2 (*10^6^) | | | Day8 (*10^6^) | | |
| --- | --- | --- | --- | --- | --- | --- |
|  | Donor #1 | Donor #2 | Donor #3 | Donor #1 | Donor #2 | Donor #3 |
| CAR1-NK | 4.9 | 4.5 | 3.6 | 65.4 | 64.1 | 69.1 |
| CAR2-NK | 4.2 | 2.4 | 2.2 | 63.0 | 57.3 | 67.5 |
| CAR3-NK | 5.0 | 3.5 | 4.7 | 68.1 | 54.6 | 72.2 |
| CAR4-NK | 3.1 | 2.6 | 2.9 | 41.7 | 43.5 | 46.2 |
| CAR5-NK | 6.0 | 6.1 | 2.8 | 54.4 | 73.2 | 66.0 |
| CAR6-NK | 5.3 | 3.1 | 3.9 | 74.0 | 59.5 | 91.3 |
| CAR7-NK | 5.1 | 4.8 | 4.0 | 58.5 | 68.2 | 69.7 |

**Table S2**. Analysis of significant differences in specific cytotoxicity between CAR1 and other CARs.

| E: T Ratio | CAR2-NK | CAR3-NK | CAR4-NK | CAR5-NK | CAR6-NK | CAR7-NK |
| --- | --- | --- | --- | --- | --- | --- |
| 0.2:1 | *p* < 0.001 | *p* < 0.001 | *p* < 0.001 | *p* < 0.001 | *p* < 0.001 | *p* < 0.001 |
| 0.4:1 | *p* < 0.001 | *p* < 0.001 | *p* < 0.001 | *p* < 0.001 | *p* < 0.001 | *p* < 0.001 |
| 0.8:1 | *p* < 0.001 | *p* < 0.001 | *p* < 0.001 | *p* < 0.001 | *p* < 0.001 | *p* < 0.001 |
| 1.6:1 | NS | NS | NS | *p* < 0.001 | *p* = 0.003 | *p* = 0.004 |

* NS, not significant.

**Table S3**. Analysis of significant differences in specific cytotoxicity between CAR5 and other CARs.

| E: T Ratio | CAR1-NK | CAR2-NK | CAR3-NK | CAR4-NK | CAR6-NK | CAR7-NK |
| --- | --- | --- | --- | --- | --- | --- |
| 0.2:1 | *p* < 0.001 | *p* < 0.001 | *p* = 0.037 | *p* < 0.001 | *p* = 0.002 | NS |
| 0.4:1 | *p* < 0.001 | *p* < 0.001 | *p* = 0.029 | *p* < 0.001 | *p* = 0.008 | *p* = 0.019 |
| 0.8:1 | *p* = 0.001 | NS | *p* = 0.011 | *p* < 0.001 | *p* < 0.001 | NS |
| 1.6:1 | *p* < 0.001 | *p* = 0.005 | *p* < 0.001 | *p* < 0.001 | *p* = 0.003 | *p* = 0.026 |

* NS, not significant.

**Table S4.** Input cell number of each NK cell population for *in vivo* anti-tumor therapy.

| Group | Percent of CAR^+^ cells | Input total NK cell  numbers (*10^6^/mouse) | Equivalent CAR-NK cell  numbers (*10^6^/mouse) |
| --- | --- | --- | --- |
| Mock-NK | \ | 16.0 | \ |
| CAR1-NK | 31.2% | 16.0 | 5.0 |
| CAR2-NK | 33.2% | 15.0 | 5.0 |
| CAR3-NK | 33.9% | 14.8 | 5.0 |
| CAR4-NK | 31.9% | 15.6 | 5.0 |
| CAR5-NK | 32.0% | 15.6 | 5.0 |
| CAR6-NK | 33.8% | 14.8 | 5.0 |
| CAR7-NK | 34.5% | 14.5 | 5.0 |
